# Attachment Problems and Mentalizing Capacity Relate to Parent–Child Informant Discrepancies in Female Adolescents with Borderline Personality Disorder

**DOI:** 10.1007/s10578-024-01735-w

**Published:** 2024-07-15

**Authors:** Mie Sedoc Jørgensen, Martin Vestergaard, Emma Beck, Ole Jakob Storebø, Stig Poulsen, Erik Simonsen, Sune Bo

**Affiliations:** 1 Psychiatric Research Unit, Region Zealand, Fælledvej 6, 4200 Slagelse, Denmark; 2 Department of Child and Adolescent Psychiatry, Psychiatry Region Zealand, Smedegade 16, 4000 Roskilde, Denmark; 3 Private Practice, Roskilde, Denmark; 4 Department of Psychology, University of Southern Denmark, Odense, Denmark; 5 Department of Psychology, University of Copenhagen, Øster Farimagsgade 2A, 1353 Copenhagen, Denmark; 6 Department of Clinical Medicine, Faculty of Health and Medical Sciences, University of Copenhagen, Copenhagen, Denmark; 7 Mental Health Services, Region Zealand East, 4000 Roskilde, Denmark

**Keywords:** Borderline personality disorder, Attachment to peers, Attachment to parents, Mentalizing, Informant discrepancies

## Abstract

Parent–child informant discrepancies on psychopathology provide important knowledge on the parent–child relationship and the child’s mental health, but mechanisms underlying parent–child informant discrepancies are largely unknown. Therefore, we investigated the relationship between attachment problems and mentalizing capacity and parent–child informant discrepancies on borderline personality disorder (BPD) severity, internalizing, and externalizing pathology in a clinical sample of 91 adolescent girls with BPD and their parents. Results showed that more attachment problems to parents and peers were related to adolescents reporting more severe BPD than parents. Adolescents who described more internalizing symptoms relative to parents, reported more parental attachment problems, but enhanced peer attachment, suggesting those adolescents who do not feel recognized by their parents might turn to their friends. When parents rated adolescents higher on externalizing behaviors, the adolescent reported more attachment problems to parents and lower mentalizing capacity, indicating that this sub-group of adolescents may reflect less about how their behavior affects others.

## Introduction

Today, it is widely accepted that parent–child informant discrepancies on the child’s emotional and behavioral problems are not merely due to measurement errors, but provide clinically important knowledge about the expression of symptoms across different contexts and the dynamics of the parent–child relationship [22, 23, 46]. Accumulating evidence suggests that parent–child informant discrepancies on emotional and behavioral problems may reflect underlying attachment problems (e.g., [10, 12, 26]).

Borderline personality disorder (BPD) is characterized by instability of affect, behaviors, and relationships [42], and attachment problems are highly prevalent in BPD [16]. However, only a few studies have investigated parent–child informant discrepancies on borderline personality features in adolescents [17, 58, 59], and it remains unclear whether attachment problems and associated problems with mentalizing in adolescents with BPD are coupled to parent–child informant discrepancies on borderline personality features, internalizing symptoms, and externalizing behaviors. To our knowledge, there is currently no specific guidance on the interpretation of informant discrepancies in BPD or other mental disorders, even though collecting information from multiple informants (e.g., children, parents, teachers) is standard practice in the assessment of child and adolescent mental health. The lack of knowledge is problematic in cases where clinicians rely on reports from both the parents and the adolescent with BPD, as clinicians risk making biased or one-sided conclusions when informants disagree. Accordingly, we need empirical evidence to guide how we interpret and make use of parent–child informant discrepancies related to personality pathology.

### Parent–Child Informant Discrepancies

Three decades ago, a meta-analytic review on cross-informant correspondence of clinical child assessment concluded that the agreement on behavioral and emotional problems between children and other informants such as parents, teachers, mental health workers, observers, and peers was surprisingly small [1]. An updated meta-analysis of 341 studies of children’s mental health replicated these findings of low multi-informant agreement but showed slightly higher agreement for externalizing behaviors compared to internalizing symptoms [22]. Studies have generally observed better multi-informant correspondence for externalizing compared to internalizing pathology [1, 22, 24]. This is likely because externalizing often relates to behaviors observable to others while internalizing symptoms are not necessarily accessible to others [22]. However, parent–child informant discrepancies are not merely explained by how obvious behaviors or symptoms appear, but also, and more importantly, reflect how the child’s experiences are shared and perceived within the parent–child dyad. Various studies have shown that higher parent–child informant discrepancies on both externalizing behaviors and internalizing symptoms are associated with more family conflict and might be associated with child maladjustment (e.g., [20, 39, 48, 50]).

### Attachment and Mentalizing Capacity as Underlying Mechanisms

As a natural part of human development, children will seek their primary caregivers with behaviors that evoke caretaking responses in adults, and these reciprocal behaviors promote the development of attachment [14]. Attachment security is a theoretical key concept developed to identify and understand individual differences in caregiver-child attachment. In short, *secure* attachment is seen when a child experiences a basic trust in the availability of its primary attachment figures [14, 15], which increases its propensity to approach the attachment figure and share affective states and experiences that call for co-regulation. Of importance to this study, attachment security therefore entails a higher degree of shared knowledge and understanding of the child’s mental states within the parent–child dyad. In contrast, *insecure* attachment takes the form of three distinct attentional, emotional, and behavioral patterns, the *avoidant*, the *anxious-resistant/ambivalent,* and the *disorganized/disoriented* attachment pattern [5], which all entail a poorer quality and quantity of shared knowledge and understanding of the child’s mental states. For example, in the insecure-avoidant attachment dyads, the child’s communication of negative affect is restricted and attention to attachment needs is deactivated to avoid experiences of the caregiver responding to vulnerability with dismissal and distancing typical of caregivers following this pattern [4]. Attachment insecurity, therefore, is likely to lead to low convergence on multiple informant reports [12, 20].

So far, only a few studies have investigated the relationship between attachment and parent–child informant discrepancies. A study by Penner et al. [49] found that insecure compared to secure attachment was related to greater discrepancy on parenting practices in a clinical sample with various mental disorders. Another study observed that community-dwelling adolescents who displayed insecure attachment reported greater parent–child discrepancies on internalizing symptoms and externalizing behaviors compared to securely attached adolescents [10]. Similarly, more adolescent-rated secure attachment has been associated with fewer parent-adolescent discrepancies on adolescent depressive symptoms, externalizing behaviors, and parent-adolescent conflict [26]. Finally, in insecurely attached inpatient adolescents with various mental disorders, parent-youth convergence on internalizing symptoms was low, but agreement on externalizing behaviors was high relative to adolescents who were securely attached [12]. Together, these findings provide compelling evidence that attachment problems could underlie parent–child discrepancies.

Fonagy et al. [30] proposed that when primary caregivers have a well-developed capacity to think about their own minds and those of others, this promotes the same mental capacity in the child. Whereas attachment styles provide information about the propensity of dyadic co-regulation of affect, mentalizing regards the capacity of using mental state information in order to make sense of each other and ourselves [7], and accordingly understand and communicate mental states correctly [33]. Impaired mentalizing capacity can lead to a lack of insight into one’s own and others’ experience and is therefore likely implicated in parent–child informant discrepancies. For instance, De Los Reyes et al. [21] found that lower emotion recognition performance (i.e., the ability to identify others’ emotions) was significantly related to greater parent and adolescent discrepant beliefs about daily life topics among a community sample of adolescents. In another study of an inpatient sample of adolescents, Borelli et al. [12] examined whether mentalizing capacity was associated with parent–child informant convergence, and observed that low adolescent self-focused reflective functioning (i.e., mentalizing that is focused on one’s own thoughts and feelings compared to others’ thoughts and feelings) was associated with higher discrepancies in parent-adolescent reports on internalizing symptoms, whereas low adolescent global mentalizing capacity was associated with high convergence in parent-adolescent reports of externalizing symptoms [12].

To summarize, both attachment and mentalizing capacity seem to be related to parent–child informant discrepancies, because high convergence could be the result of shared parent–child reflections on the adolescents’ thoughts and feelings, and/or the result of better communication between the child and the caregiver within the context of a secure attachment relationship.

### Parent–Child Informant Discrepancies in Borderline Personality Disorder Research

The etiology of BPD is theorized to evolve out of bidirectional interactions between inherent biological vulnerabilities that can challenge sensitive caring [32], and an invalidating environment [43]. Consequently, there may be instances where the adolescent with BPD has difficulties in adequately mentalizing his or her own and others’ behaviors and intentions, where parents may hold more accurate perceptions. On the other hand, there might be circumstances where the environment is characterized by invalidation and adverse parenting styles, in which the parent lacks the ability to adequately understand, mirror, and reflect upon the emotions and behaviors of their child, which can lead to an undermining of the child’s views or to oversimplifying or minimizing problems. In these cases, the child may hold more accurate reflections of own mental state.

Adolescents with BPD show marked dysfunctions in mentalizing as well as attachment difficulties [6, 13, 17, 45]. However, relatively few studies have investigated parent–child informant discrepancies in BPD, and the results from these studies differ. For instance, some studies find high concordance between parents and adolescents with regard to presence of BPD diagnosis and/or BPD severity [17, 57–59]. In contrast, other studies have found that adolescents generally rate themselves higher on BPD features than their parents [58], while others found the opposite relationship [59]. These studies, however, converge in finding that agreement on externalizing behaviors generally is higher compared to internalizing symptoms and emotional and identity-related BPD features such as fear of abandonment and emptiness [17, 57]. Vanwoerden et al. [57] found that for parents and female adolescents, attachment security predicted lower levels of BPD severity in the consensus factor, which means that insecure attachment was related to how parents and female adolescents collectively viewed BPD. Lastly, the same study found that high parent-adolescent consensus on BPD severity predicted lower mentalizing ability in adolescents, which is consistent with findings that increased psychopathology is associated with reduced mentalizing capacity [57].

To sum up, there are only few studies on parent–child informant discrepancies within the field of BPD research, and in particular in samples with diagnosed BPD. Of these, only few have investigated possible mechanisms behind parent–child informant discrepancies.

### Aims

The current study aimed to explore parent–child informant discrepancies on the severity of BPD features, externalizing behaviors, and internalizing symptoms in a sample of female adolescents with BPD. Moreover, we aimed to explore whether adolescents’ self-reported mentalizing capacity and attachment problems were coupled with parent–child informant discrepancies. We will test two hypotheses; namely whether; (1) adolescent attachment problems and (2) impaired adolescent mentalizing capacity are related to increased parent–child informant discrepancies on the severity of BPD features, externalizing behaviors, and internalizing symptoms.

## Methods

### Design

#### Sample and Procedures

The study sample consisted of 91 females and one of the parents of each of these. Parental reports were predominantly completed by mothers (n = 70, 77%; fathers: n = 21, 23%). Participants and their parents were recruited from four child and adolescent psychiatric outpatient clinics in Region Zealand, Denmark, who attended baseline psychiatric assessments prior to participation in a randomized controlled trial [8], ClinicalTrials.gov identifier: NCT02068326). Informed consent was obtained from the adolescents and their caregivers. Inclusion criteria for the trial included the following: (1) ages 14 to 17 years, (2) meeting a minimum of four DSM-5 BPD criteria, and (3) having a total score above clinical cut-off (> 67) on the Borderline Personality Feature Scale for Children (BPFS-C; [18]). Exclusion criteria included the following: (1) current psychosis or diagnosis of schizophrenia or schizotypal personality disorder, (2) comorbid diagnosis of pervasive developmental disorder, (3) learning disability, (4) anorexia, (5) antisocial personality disorder, (6) current (past two months) substance dependence, (7) BPD was not considered the primary diagnosis, and lastly (8) current psychiatric inpatient treatment [8]. For the current study, we excluded dyads that did not include biological parents (i.e., parent substitutes, n = 20). All procedures were approved by the Regional Ethics Committee of Zealand (no: SJ-371), and the data is registered at the Danish Data Protection Agency (no: REG-55–2014).

#### Measures

All participants were thoroughly assessed using semi-structured interviews and self-reported measures. Mental disorders were assessed with the Mini-International Neuropsychiatric Interview for children and adolescents (MINI-KID 6.0; [53]). BPD was assessed with the Childhood Interview for DSM-IV Borderline Personality Disorder (CI-BPD, [60]). PDs were assessed using the Structured Clinical Interview for DSM-IV-Axis II (SCID-11; [29]). After the diagnostic interviews, participants and parents filled out questionnaires, all of which were applied in Danish-translated versions. We used published Danish versions of the Youth Self Report and the Child Behavior Checklist [11, 38]. The remaining measures were translated and back-translated following standard procedures.

#### Parent–Child Informant Measures

*Borderline personality features* were measured with the Borderline Personality Features Scale for Children (BPFS-C; [18]) and the correspondent parent version the Borderline Personality Features Scale for parents (BPFS-P; [52]). The BPFS is a 24-item self-report instrument used to assess borderline personality features in children and adolescents*.* It consists of four subscales with six items per subscale: affective instability (e.g., “My feelings are very strong. For instance, when I get mad, I get really really mad. When I get happy, I get really really happy”), identity problems (e.g., “I change my mind almost every day about what I should do when I grow up”), negative relationships (e.g., “People who were close to me have let me down”), and self-harm (e.g.,, “I do things that other people consider wild or out of control”). Items are rated on a 5-point Likert scale ranging from 1 (not at all true) to 5 (always true). Internal consistency on the BPFS-C total scale and subscale was fair to excellent (Cronbach’s α were as follows: 0.84 (total scale), 0.69 (affective instability), 0.61 (identity problems), 0.63 (negative relationships, and 0.74 (self-harm). Internal consistency was good to excellent for the BPFS-P. Cronbach’s α were as follows: 0.87 (total scale), 0.83 (affective instability), 0.56 (identity problems), 0.62 (negative relationships, and 0.78 (self-harm).

*Internalizing and externalizing* within the last six months were measured with the 112-item Youth Self Report (YSR; [2]) and the corresponding parental version, the Child Behavior Checklist (CBCL; [3, 11, 38]). Internalizing included the Anxious/Depressed, Withdrawn/Depressed, and Somatic Complaints subscales, and Externalizing included the Rule-breaking and Aggressive Behavior subscales. Items are rated on a 3-point Likert scale ranging from 0 (not true) to 2 (very true or often true). Internal consistency for the YSR was found to be good to excellent (Cronbach’s alpha for internalizing and externalizing: 0.86 and 0.83, respectively), and internal consistency for the CBCL was also found to be good to excellent (Cronbach’s alpha for internalizing and externalizing: 0.83 and 0.90, respectively).

#### Self-Reported Attachment and Mentalizing Capacity

*Attachment problems* were measured with the 53-item Inventory of Parent and Peer Attachment-Revised (IPPA-R; [36]). The IPPA-R uses a 3-point Likert scale and consists of two subscales: (1) attachment to parents and (2) attachment to peers in youth. It measures the quality of communication, feelings of trust, and degree of alienation (e.g., “My parents/friends can tell when I’m upset about something”, “When we discuss things, my parents/friends care about my point of view” and “My parents/friends help me to understand myself better”). Internal consistency for this measure was found to be excellent (Cronbach’s alpha for both subscales: 0.94).

*Mentalizing capacity* was measured with the 46-item self-report Reflective Function Questionnaire for Youth (RFQ-Y; [37]). The RFQ-Y measures adolescent reflective functioning on a 6-point Likert scale (e.g.,”I find it difficult to see other people’s point of view”, “Strong feelings often cloud my thinking”, “I like to think about the reasons behind my actions”). The measure consists of two subscales A and B, and a total score, which was used in the present study. Internal consistency was found to be acceptable (Cronbach’s alphas of 0.69 and 0.70 for subscales A and B, respectively, and 0.57 for the total scale).

#### Statistical Analyses

Statistical analyses were conducted in SPSS 28. We calculated a discrepancy ratio score for the YSR/CBCL internalizing and externalizing scales and for the BPFS-C/BPFS-P total scale. To ensure that associations were not simply explained by differences in symptom severity, we used a normalized ratio score ((adolescent–parent) / (adolescent+parent)), which corresponds to using a standardized difference score [19], but limits unnecessary transformation. A *p*-value of < 0.05 was considered significant.

As a first step, we used bivariate Pearson correlations to investigate relationships between the parent and youth reports on the CBCL/YSR internalizing and externalizing scales, and the BPFS scale. We then used paired-samples t-tests to compare the mean differences in the parent relative to adolescent scores on the YSR/CBCL externalizing and internalizing scales and the BPFS total scale as well as the four subscales. We used independent two-tailed t-tests to determine whether the included dyads differed from study dropouts on the CBCL/YSR and the BPFS.

To test our hypotheses that more attachment problems and lower mentalizing capacity were associated with increased parent–child informant discrepancies on the YSR/CBCL and the BPFS-C/BPFS-P, we used multiple linear regression analyses. To limit the amount of testing and to target general BPD severity, we specifically explored the total scale of the BPFS and not the four subscales. The discrepancy ratio measures were entered as the dependent variable in separate multiple linear regression models. The IPPA-R Parents, IPPA-R Peers, and RFQ-Y were entered together in the model as the covariates of interest, controlling for age, and the other discrepancy ratio measures to assure that findings were independent of each other.

Continuous variables were determined to have a significantly non-normal distribution if the standard errors of the skewness were above or below Z ± 2.57 (two-sided p < 0.01). The baseline IPPA-R Peers score was significantly non-normally distributed and was therefore normalized with Rankit transformation. The remaining variables all appeared normally distributed. Multicollinearity was not a problem with a tolerance > 0.3.

## Results

### Descriptive Statistics

See Table 1 for characteristics of the participants. Eighty-nine participants (98%) met diagnostic criteria for BPD (≥ 5) on the CI-BPD and two participants (2%) fulfilled four DSM-5 BPD criteria. Co-occurring mental disorders were prevalent. The mean scores on the BPFS-C and the BPFS-P were above clinical cut-off of 66 (BPFS-C: M = 79.8, *SD* = 12.0; BPFS-P: M = 77.2, *SD* = 13.9). Internalizing symptoms were in the clinical range on the YSR (M = 32.5, *SD* = 9.6) and the CBCL (M = 25.3, *SD* = 9.1), and externalizing behaviors were in the borderline clinical range on the YSR (M = 25.8, SD = 8.2) and the CBCL (20.5, *SD* = 11.4). To the authors’ knowledge, there are no clinical cut-offs for the RFQ-Y or the IPPA-R, but with a mean score of 51.4 (*SD* = 11.43) on the IPPA-R Parents scale, our sample scored more than three *SD*s above the level of the level seen among Gullone and Robinson’s [36] non-clinical sample of adolescents (M = 21.7, *SD* = 8.71), indicating that our sample reported considerably more attachment problems with their parents. Similarly, our sample scored a mean of 42.3 (*SD* = 9.66) on the IPPA-R Peers scale, which is around two *SD*s above the level seen in Gullone and Robinson’s [36] non-clinical sample of adolescents (M = 26.5, *SD* = 7.94). Lastly, our sample scored a mean of 7.66 (*SD* = 0.59) on the RFQ-Y, which is more than one *SD* below a mixed clinical inpatient sample in Ha et al.’s [37] study (M = 8.73, *SD* = 0.92), indicating greater impairments in mentalizing than among adolescent inpatients.

**Table 1:** Characteristics of the participants

	N = 91 (n, %)
Age (M, *SD*)	15.8 (1.01)
Attends school	77 (85)
Parental alcohol/substance abuse	31 (34)
Number of BPD criteria (M, *SD*)	7.4 (1.41)
SCID-II^a^	
Paranoid	38 (41)
Narcissistic	1 (1)
Obsessive–compulsive	16 (18)
Avoidant	29 (32)
Dependent	9 (10)
MINI-KID	
Major depressive disorder	85 (94)
Panic disorder	38 (42)
Agoraphobia	64 (70)
Social phobia	51 (56)
Generalized anxiety disorder	21 (23)
Post-traumatic stress disorder	8 (9)
Obsessive–compulsive disorder	28 (31)
Attention-deficit hyperactivity disorder	16 (18)
Conduct disorder	16 (18)
Oppositional defiant disorder	35 (39)
Alcohol problems	24 (26)
Substance use problems	14 (15)
Mean number of MINI-KID disorders (M, *SD*)	4.4 (1.84)
IPPA-R Parents	51.4 (11.43)
IPPA-R Peers	42.3 (9.66)
RFQ-Y	7.66 (0.59)

*NB: SCID-II* Structured Clinical Interview for DSM-IV-Axis II; *MINI-KID* Mini-International Neuropsychiatric Interview for children and adolescents; *IPPA-R* Inventory of Parent and Peer Attachment-Revised; *RFQ-Y* Reflective Function Questionnaire for Youth

^a^No occurrences of schizoid, schizotypal, and histrionic personality disorder

See Table 2 for the results of the correlational analyses on our key study variables. Regarding the head-to-head comparisons of parent–child variables, we found that 1) YSR internalizing correlated with CBCL internalizing, 2) YSR externalizing correlated with CBCL externalizing, and lastly, 3) on the BPFS, we only found a significant correlation on the affective instability subscale.

**Table 2:** Correlational table of key study variables

Measure	CBCL int	CBCL ext	BPFS-P total	BPFS-P affective	BPFS-P identity	BPFS-P relationships	BPFS-P self-harm
YSR int	0.382^**^	− 0.104	− 0.109	− 0.141	− 0.020	− 0.078	− 0.096
YSR ext	− 0.003	0.416^**^	0.314^**^	0.245^*^	0.088	0.216^*^	0.413^**^
BPFS-C total	0.113	0.071	0.123	0.064	0.125	0.043	0.161
BPFS-C affective	0.048	0.323^**^	0.327^**^	0.373**	0.191	0.129	0.304^**^
BPFS-C identity	0.086	− 0.134	− 0.013	− 0.113	0.122	− 0.004	− 0.009
BPFS-C relationships	0.166	− 0.055	0.091	− 0.013	0.149	0.175	0.031
BPFS-C self-harm	0.004	0.024	− 0.002	− 0.025	− 0.016	− 0.140	0.142

*NB: YSR* Youth Self Report; *CBCL* Child Behavior Checklist, *BPFS* Borderline Personality Features Scale; *IPPA-R* Inventory of Parent and Peer Attachment-Revised; *RFQ-Y* Reflective Function Questionnaire for Youth

^**^significant at the 0.01 level (two-tailed)

^*^significant at the 0.05 level (two-tailed)

Paired samples t-tests (cf. Table 3) revealed that there were significant parent–child differences on the YSR/CBCL externalizing, on the YSR/CBCL internalizing, and lastly on the identity problems subscale of the BPFS.

**Table 3:** Significant differences between parents and adolescents on study variables

Variable		Adolescents (n = 91)	Parents (n = 91)	*t*-value	*df*	Sig (two-tailed)
Externalizing	M *SD*	25.8 (8.2)	20.5 (11.4)	4.566	90	< .001
Internalizing	M *SD*	32.5 (9.6)	25.3 (9.1)	6.578	90	< .001
BPFS total	M *SD*	79.8 (11.8)	77.2 (13.8)	1.417	90	0.16
BPFS affective	M *SD*	20.0 (3.0)	20.9 (5.0)	− 1.805	90	0.074
BPFS identity	M *SD*	20.3 (4.0)	19.2 (3.7)	2.112	90	0.037
BPFS relationships	M *SD*	18.3 (3.1)	18.1 (3.7)	0.426	90	0.67
BPFS self-harm	M *SD*	18.8 (3.6)	19.0 (4.7)	− 0.305	90	0.76

*NB: BPFS* Borderline Personality Features Scale

### Parent–Child Discrepancy Analyses

See Table 4 for an overview of the multiple linear regression models. The multiple linear regression analysis showed that an increased BPD feature discrepancy score was significantly associated with a higher IPPA-R Parents score (p = 0.030) and IPPA-R Peers score (p = 0.007), suggesting that adolescents who rated themselves higher on BPD features than the parents reported more attachment problems to both parents and peers.

**Table 4:** Associations between parent–child discrepancies, attachment problems, and mentalizing capacity

	B	SE	β	t	P
Dependent variable: Parent–child discrepancy on BPFS					
IPPA-R Parents	0.002	0.001	0.158	2.212	0.030
IPPA-R Peers	0.023	0.008	0.197	2.781	0.007
RFQ-Y	− 0.018	0.013	− 0.094	− 1.402	0.165
Dependent variable: Parent–child discrepancy on Internalizing					
IPPA-R Parents	0.003	0.002	0.193	2.122	0.037
IPPA-R Peers	− 0.042	0.019	− 0.206	− 2.248	0.027
RFQ-Y	− 0.038	0.029	− 0.111	− 1.302	0.196
Dependent variable: Parent–child discrepancy on Externalizing					
IPPA-R Parents	− 0.006	0.002	− 0.257	− 3.753	< 0.001
IPPA-R Peers	0.013	0.021	0.044	0.595	0.553
RFQ-Y	0.076	0.032	0.158	2.395	0.019

*NB: IPPA-R* Inventory of Parent and Peer Attachment-Revised; *RFQ-Y* Reflective Function Questionnaire for Youth

All linear regression models were controlled for age, and the other parent–child discrepancy scores not entered as the dependent variable. Model degrees of freedom = 83

The regression analysis showed that a higher internalizing discrepancy score was significantly associated with a higher IPPA-R Parents score (p = 0.037), but a lower IPPA-R Peers score (p = 0.027). Thus, adolescents who rated themselves proportionally higher on internalizing symptoms compared to the parents had worse attachment to parents but in contrast better attachment to their peers.

Finally, a higher externalizing discrepancy score was significantly associated with a lower IPPA-R Parents score (p < 0.001), and a higher RFQ-Y score (p = 0.019). These results show that those adolescents who rated themselves lower on externalizing symptoms relative to their parents reported more attachment problems to the parents, and at the same time, these adolescents rated themselves as having more impaired mentalizing capacity.

## Discussion

The primary aim of the current study was to investigate whether and how attachment problems and/or mentalizing capacity were related to parent–child informant discrepancies among adolescents with BPD. In line with previous studies (e.g., [12, 26]), the results showed that increased parent–child discrepancies on BPD severity and both internalizing symptoms and externalizing behaviors were significantly associated with more attachment problems. Specifically, we found that more attachment problems to parents and peers were related to increased parent–child discrepant views on BPD severity with the adolescents reporting more BPD features than the parents. Furthermore, more attachment problems to parents, but lower attachment problems to peers, were associated with adolescents reporting more internalizing symptoms relative to parents. This suggests that adolescents with increased internalizing symptoms, which are not recognized by their parents, experience themselves as having better attachment to their friends. Our findings that attachment problems and discrepancy on internalizing symptoms are related, fit well with a model in which parent–child attachment insecurity and sub-optimal co-regulation of affect might make a child less inclined to communicate openly about mental states. This could potentially leave the child with higher risk of developing affect regulation difficulties and parents less knowledgeable about the subjective state of the child. Consequently, the child may turn to their peers for regulation of affect, which could explain why higher parent–child discrepancies on internalizing symptoms were associated with less attachment problems with peers in the present study.

A different relationship was found for externalizing behaviors, where more attachment problems with parents were associated with increased parent-adolescent disagreement on externalizing behaviors, driven by parents rating the adolescents higher on externalizing than the adolescents themselves, which is in line with previous studies (e.g., [26]). At the same time, however, adolescents who described fewer externalizing behaviors relative to their parents, reported lower mentalizing capacity, indicating that those adolescents may hold a less accurate evaluation of how their behavior is perceived by others.

Regarding parent–child discrepancy on BPD severity, we found that increased discrepancy was related to more attachment problems with peers. It is possible that this finding is a reflection of the close relationship between BPD and attachment difficulties, which means that when adolescents rate themselves with more severe BPD, they will also rate themselves highly on attachment problems. When inspecting the items on the “negative relationships” subscale of the BPFS, there is an overlap with the items on the IPPA-Peers scale. However, this finding should be corroborated in future studies.

These findings on the relationship between attachment and parent–child informant discrepancies have important implications, since higher discrepancies on internalizing symptoms and externalizing behaviors are associated with more family conflict and child distress [20, 39, 48, 50]. Parental stress related to the high burden of care in BPD [41] and parental psychopathology [40] are both associated with an increased risk of bias in parents’ perception of their child’s emotions and behavior (e.g., [9, 44, 56]). This may play an important role for the intragenerational transmission of insecure attachment [27], which is of specific relevance to the development of BPD [31].

Concerning mentalizing capacity, we found that adolescents who reported less externalizing behaviors relative to their parents reported lower mentalizing capacity. This finding was, at first sight, surprising since externalizing psychopathology, in general, relates to impairments in mentalizing [54, 55]. Specifically, mentalizing entails the process of using mental state information in order to explain behaviors [31], and accordingly a more flexible interpretation of one’s own and others’ behaviors, which should serve as a buffer that inhibits impulsive and aggressive behaviors [47]. Therefore, we would expect that more externalizing behaviors rated by the adolescents would be associated with lower adolescent-rated mentalizing capacity. A possible explanation for this finding could be that parents are better raters of externalizing behaviors than the adolescents, because 1) parent scores on externalizing and BPD severity are highly correlated, and 2) BPD and mentalizing are related constructs. Accordingly, when parents see more externalizing behaviors than the adolescents, it could be because BPD severity is increased and consequently the mentalizing capacity of the adolescent is lower, and consequently that the adolescents with impaired mentalizing may not be the best raters of their externalizing behaviors. Since lower adolescent- compared to parent-reported externalizing at the same time was associated with more attachment problems and lower mentalizing capacity, another possibility is that adolescents with attachment problems may reflect less about what impact their behavior and actions have on others. Note that the parent–child discrepancy analyses do not show that adolescents who reported more externalizing behaviors had better mentalizing capacity but rather show the relative perception the adolescents had of their externalizing behaviors compared to the parents.

Similar to previous studies on BPD (e.g., [17, 57]), we did not find a significant difference between the parents’ and adolescents’ mean scores on the BPFS, which indicates that they agree about the severity of BPD. However, the BPFS scores were only significantly correlated for the subscale affective instability. A study of Danish adolescents shows that affective instability is the BPD feature that has the most explanatory value when comparing healthy adolescents to adolescents with BPD [34] in progress. Accordingly, affective instability might be a core BPD feature that is more recognizable to both the adolescents and the parents. Despite no correlation between the BPFS total scale and the subscales negative relationships, identity problems, and self-harm, the adolescents and the parents only scored significantly different on the subscale identity problems, which we speculate might be related to the abstract and subjective nature of the concept identity. Regarding externalizing behaviors and internalizing symptoms, the adolescents scored significantly higher than their parents, but parent and adolescent scores were significantly correlated.

Our results have important clinical implications. Our findings show that parent–child informant discrepancies in BPD provide meaningful information central to the targets of family intervention for BPD in adolescence, attachment security, and parent–child co-regulation of affect. Informant discrepancies, therefore, are relevant to include in the assessment, psychoeducation, and treatment of adolescents with BPD. In the assessment of adolescents with BPD, clinicians must carefully consider information from both parents and adolescents to comprehensively understand the adolescent’s symptomatology. When parents and adolescents disagree, clinicians should assess for attachment problems and mentalizing capacity, because discrepancies may signal dysfunctions in these underlying mechanisms.

Clinicians might consider using the discrepant views when providing psychoeducation about BPD to the parents and the adolescents as a means to scaffold mentalizing by encouraging both parents and adolescents to mentalize one another. Helping the parents to better understand the internal suffering of their child, and the adolescents to more effectively verbally express what they are experiencing could facilitate collaborative treatment planning and increase treatment outcomes. Acknowledging the significant impact of parental perceptions on treatment outcomes, it is furthermore important that clinicians pay attention to potential shifts in perceived severity, particularly as parents and adolescents gain insight into each other’s perspectives on the adolescent’s pathology through psychoeducation and family-based interventions. For instance, a parent’s lack of awareness pre-treatment about a symptom that the adolescent experiences may result in a relative increase in perceived severity of that symptom in the first treatment sessions. This apparent increase in symptom severity may not reflect deterioration of the adolescent’s mental health but rather that either the parent or the adolescent has gained insight into the symptoms that the adolescent experiences.

Building upon our findings, interventions for families with high levels of discrepancy between parent and child views may consider providing comprehensive psychoeducation to both parents and adolescents at the outset of treatment. Second, treatment could include family therapy with a focus on the dynamics within the parent–child dyad. In particular, integrating attachment-focused interventions such as mentalization-based treatment for adolescents might increase treatment outcomes.

In treating families characterized by high discrepancy, clinicians should consider the specific concerns raised by the parents and the children, because understanding the different perspectives might help tailor more effective treatments, promote mentalization and co-regulation of affect within the parent–child dyad, and prevent the negative consequences associated with higher parent–child discrepancies. Previous studies have found that high parent–child discrepancies predict poorer treatment outcomes in children with various psychiatric diagnoses [35], and also predict future delinquent behavior [28]. Accordingly, targeting discrepant views should be an important treatment goal. The current study adds to accumulating evidence that clearly points to the importance of taking discrepant parent-adolescent views seriously as these may reflect underlying issues that can worsen adolescent mental health problems.

## Limitations

There are several important limitations to the current study. One major limitation has to do with the fact that we do not have parental measures of attachment and mentalization, and therefore solely rely on the adolescent reports, which furthermore is self-report and not assessed through a structured clinical interview such as the Child Attachment Interview [51], or Movie for the Assessment of Social Cognition [25]. Moreover, the IPPA-R does not look at specific attachment styles, but only severity of attachment problems. The sample was a clinical sample with severe BPD, which limits the generalizability to adolescents with less severe or subthreshold BPD. Furthermore, the study sample consisted exclusively of female adolescents, and results should, accordingly, not be generalized to male adolescents. Likewise, mothers constituted almost 80% of the sample, and the study was underpowered to look for differences between, for instance, mothers and fathers. The current study used a measure of BPD that is based on the categorical BPD diagnosis. Developing parent–child measures based on dimensional personality assessment, including personality functioning, that go beyond the categorical BPD diagnosis could further expand this area and elucidate how specific traits influence parent–child perceptions and dynamics.

## Conclusions

In a clinical sample of adolescents with BPD and their parents, we showed that attachment problems are associated with parent–child informant discrepancies on externalizing behaviors, internalizing symptoms, and BPD features. Mentalizing capacity, on the other hand, only appeared to be associated with parent–child informant discrepancies on externalizing behaviors, where higher discrepancies were related to higher adolescent-rated mentalizing capacity. These findings have important clinical implications concerning the assessment of BPD because when parents and children disagree on the child’s mental state, the clinician should assess whether there are underlying attachment problems and impairments in mentalizing. Furthermore, our findings have implications with regard to treatment planning as increased parent–child discrepancies suggest that therapy needs to target the dynamics in the parent–child and child-peer relationships, so the parents are able to better support their children towards recovery. This study adds to previous findings that highlight the importance of not dismissing the validity of multi-informant reports and warrants the clinicians’ attention in cases where the informants disagree because the discrepancies offer important information about the parent–child relationship.

## Summary

In a clinical sample of 91 adolescent girls with BPD and their parents, the current study finds significant associations between attachment problems and informant discrepancies. Adolescents with more attachment problems to parents and peers report more severe BPD than their parents. Those adolescents who report more internalizing symptoms relative to their parents also report more parental attachment problems but show enhanced peer attachment, suggesting a shift in emotional support from parents to friends. Additionally, when parents rate their children higher on externalizing behaviors, the adolescents report more attachment problems with their parents and at the same time demonstrate lower mentalizing capacity, implying a lower reflection on the impact of their behavior on others.

The study concludes that attachment problems are intricately linked to parent-child informant discrepancies across externalizing behaviors, internalizing symptoms, and BPD features. Mentalizing capacity was only associated with discrepancies in externalizing behaviors. Clinically, these findings underscore the importance of considering attachment problems and mentalizing impairments when assessing BPD from multiple informants. Discrepancies between parent and child reports should not be overlooked, as they provide critical insights into the dynamics of the parent–child relationship. The study reinforces the value of multi-informant reports and highlights the necessity for clinicians to investigate the underlying causes of informant discrepancies to enhance assessment and for effective treatment planning, emphasizing the need to address both parent-child and child-peer relationships to support adolescents with BPD towards recovery.

## Data Availability

Anonymized data is available on request. Requests for data should be directed to the Primary Investigator, Professor Erik Simonsen, es@regionsjaelland.dk.
